# Evidence of spontaneous mentalizing in children with Cornelia de Lange and fragile X syndromes, but not autistic children

**DOI:** 10.1093/oons/kvaf003

**Published:** 2025-10-23

**Authors:** Katherine Ellis, Jo Moss, Malwina Dziwisz, Beth Jones, Christina Griva, Sophie Pendered, Roisin C Perry, Sarah J White

**Affiliations:** Clinical, Educational and Health Psychology, University College London, 1-19 Torrington Place, London WC1E 7HB, United Kingdom; School of Psychology, University of Surrey, Guildford GU2 7XH, United Kingdom; School of Psychology, University of Surrey, Guildford GU2 7XH, United Kingdom; National Hospital for Neurology and Neurosurgery, Queen Square, London WC1N 3BG, United Kingdom; School of Psychology, University of Surrey, Guildford GU2 7XH, United Kingdom; School of Psychology, University of Surrey, Guildford GU2 7XH, United Kingdom; Walton Centre, Lower Lane, Fazakerley, Liverpool L9 7LJ, United Kingdom; Institute of Education, University College London, 20 Bedford Way, London WC1H 0AL, United Kingdom; Institute of Cognitive Neuroscience, University College London, Alexandra House, 17-19 Queen Square, London WC1N 3AZ, United Kingdom

**Keywords:** Cornelia de Lange syndrome, fragile X syndrome, false belief, mentalizing, intellectual disability, autism

## Abstract

It has been suggested that mentalizing abilities underlie the distinct profiles of autism characteristics observed between Cornelia de Lange (CdLS) and fragile X syndromes (FXS) and autistic people without a genetic syndrome. However, traditional explicit mentalizing tasks have high language demands that may mask true mentalizing abilities in these populations. We compared performance on traditional explicit tasks and an implicit anticipatory looking mentalizing task in children with CdLS (N = 9), boys with FXS (N = 9), autistic (*N* = 22) and neurotypical (*N* = 34) children. The groups showed divergent patterns of performance. Neurotypical children had higher explicit mentalizing scores than all other groups. However, neurotypical, FXS and CdLS groups showed better implicit mentalizing performance than autistic children. Both chronological age and receptive language ability correlated with explicit mentalizing scores in neurotypical children. In autistic children, there was an association between explicit mentalizing score and receptive language ability but not chronological age. Explicit mentalizing score was not associated with receptive language ability or chronological age in the CdLS and FXS groups. Neither chronological age nor receptive language ability correlated with implicit mentalizing task performance in any group. Findings suggest that explicit tasks may mask true mentalizing abilities in autistic children, children with CdLS and children with FXS.

## INTRODUCTION

Autism spectrum disorder (ASD)^1^ is characterised by the presence of persistent differences in social communication and interaction, alongside restricted and repetitive behaviours, interests or activities [1]. Many genetic syndromes associated with intellectual disability (ID) are more likely to show high levels of autism characteristics [2]. Yet, these groups show highly heterogeneous profiles of autism characteristics, that may be distinct, in very subtle ways, from those observed in autistic people without a genetic syndrome [3]. Like autistic people, people with genetic syndromes also show atypical development of social cognitive abilities [4], defined here as the cognitive abilities involved during social interaction [5]. A key social cognitive ability thought to contribute specifically to the development of autism characteristics in autistic people without a genetic syndrome is mentalizing [6].

Mentalizing is the ability to understand and reason about one’s own and others’ mental states [7]. However, the role of mentalizing in the development of autism characteristics within genetic syndromes, and the extent to which it may account for variable profiles in social-communication skills between these populations and autistic people without a genetic syndrome is not well understood. Here, we present a study investigating mentalizing between children with two such genetic syndromes, Cornelia de Lange (CdLS) and fragile X syndromes (FXS), alongside autistic and neurotypical children. Comparisons between these syndromes and autistic children without genetic syndromes enables investigation of which social cognitive mechanisms may be shared or distinct between these groups. Improved knowledge of the similarities and differences of social-cognitive factors that underpin variation in autism social and communication characteristics between children in these syndrome groups and autistic children, can guide targeted support and inform autism assessment and diagnosis in autistic people with and without a genetic syndrome. Such work will also provide insight into the mechanistic pathways from genes, to neurobiology, to cognition, to behaviour across these neurodevelopmental conditions (Ellis et al., in prep).

CdLS and FXS are two genetic syndromes chosen for their high levels of autism characteristics [2], particularly social and communication characteristics [8]. Critically, these syndromes evidence a high degree of specificity in relation to the presentation of these characteristics relative to people with other genetic syndromes associated with ID and autistic people without a genetic syndrome [3]. Both people with CdLS and FXS have high levels of autism reciprocal social interaction characteristics, while those with CdLS show greater levels of autism communication differences. Further refinement of the behavioural phenotype in CdLS, using item-level scores from the Social Communication Questionnaire [9], indicates that while there is a great deal of overlap of scoring profiles between people with CdLS and autistic people without a genetic syndrome, those with CdLS are less likely to evidence autism-related differences in very specific aspects of social-communication, such as repetitive and stereotyped speech, use of gestures and social smiling, relative to autistic people without a genetic syndrome [10]. Likewise, whilst boys with FXS broadly show a similar (albeit lesser) profile of autism characteristics relative to autistic children without a genetic syndrome, item-level analysis of the Autism Diagnostic Observation Schedule [11] indicates specific advantages in gaze integration, quality of social overtures, social smiling, facial expressions and response to joint attention [12]. Such behavioural variability observed between groups brings to question the degree to which the emergence of these profiles of social and communication characteristics results from the same mechanisms (e.g. mentalizing) that are thought to underpin similar behaviours in autistic people without a genetic syndrome. Understanding the convergence and divergence of social cognitive profiles, and the mechanisms that underpin them, between these groups and autistic people without a genetic syndrome can provide insight into their autism characteristics and inform whether autism support can benefit people with CdLS and FXS [13]. In addition, this understanding may also highlight potential genetic, biological, and cognitive mechanisms that contribute to the heterogeneity of social and communication characteristics within autistic people without a genetic syndrome [14].

However, a key challenge for comparing mentalizing abilities across these groups is the lack of appropriate tasks for those with ID. Previous work has investigated mentalizing skills in those with CdLS and FXS primarily via traditional explicit false belief tasks such as the Sally-Anne [15] or Smarties task [16]. Collectively, such studies have indicated a significant delay when these individuals pass false belief tasks relative to neurotypical controls. However, evidence suggests that people with genetic syndromes may fail explicit mentalizing tasks, due to their high language demands (e.g. requiring verbal comprehension, and a verbal response [17–19]). These findings suggest that traditional explicit mentalizing tasks may not have adequate sensitivity for populations associated with ID. In addition, these same non mentalizing skills may be recruited by some autistic people without a genetic syndrome to pass these tasks through non-mentalistic reasoning [20], indicating these are not pure tasks of mentalizing and may be less suitable for the autistic population more broadly.

Recent work has aimed to address the lack of appropriate tasks via assessment of intentionality abilities; which are early social cognitive abilities considered to be pre-cursors to the explicit mentalizing reasoning abilities assessed by traditional tasks (Ellis et al., in prep, [4, 21]). For example, Ellis and colleagues [4] engaged neurotypical and autistic children, and children with CdLS, FXS and Rubinstein-Taybi syndrome (RTS) with a developmental battery assessing early social cognitive abilities [22]; neurotypical children, and children with FXS and RTS passed significantly more tasks assessing early social cognitive abilities compared to autistic children and children with CdLS of similar non-verbal mental ages. Whilst these tasks help to unpick key alternative social cognitive trajectories between diagnostic groups, they don’t assess mentalizing abilities per se, argued by some researchers to be the core social cognitive mechanism that distinguishes non-autistic and autistic people [23].

Therefore, in the current study we utilise implicit tasks based on an anticipatory looking paradigm to assess spontaneous mentalizing; we argue that these are more sensitive tasks that also have the benefit of being non-verbal and therefore more widely accessible [24]. Spontaneous implicit mentalizing refers to the automatic, unconscious, and rapid processing of others’ mental states, which is distinguishable from explicit reasoning about others’ mental states [7]. Autistic adults who pass explicit mentalizing tasks have been found not to spontaneously mentalize during implicit tasks [25]; this finding has been replicated with autistic children [26, 27] and their younger siblings too [28]. Not only may these tasks be more suitable for populations associated with ID by virtue of having no verbal requirements, but these findings suggest that implicit measures also provide a more sensitive assessment of mentalising in autistic people without an ID. Variability in implicit measures may therefore meaningfully contribute to the subtle differences in autism social and communication profiles observed between different genetic syndromes and non-syndromic autism [10, 12]. These tasks also hold the unusual promise of a potential assessment of autism-related characteristics suitable for children and adults both with and without an ID.

Here, we present the first study to assess performance on an implicit mentalizing task, and to compare explicit and implicit mentalizing task performance between autistic children, and children with two genetic syndromes (CdLS and FXS) with heterogeneous autism characteristics. We aimed to (A1) compare performance profiles on a battery of explicit verbal story-based false belief tasks and an implicit anticipatory-looking multi-trial false belief task in children with CdLS, with FXS, and autistic and neurotypical children, and (A2) investigate whether and how age and language ability are associated with both explicit mentalizing and implicit task performance across these groups.

Based on a vast body of previous research e.g. [17, 19, 29, 30], we expected neurotypical children to show more accurate performance on explicit tasks compared to autistic children and as well as to those with CdLS and FXS. We also predicted that neurotypical children would perform better than autistic children on the implicit anticipatory-looking task, replicating previous findings in this group [26, 27]. We expected that performance on the implicit task among children with CdLS and FXS will provide insight into why these groups struggle with explicit mentalizing tasks. If difficulties with explicit task is due to mentalizing difficulties, we would expect children with CdLS and FXS to show less accurate implicit mentalizing task performance compared to neurotypical children. However, if difficulties with explicit performance is due to other task demands (e.g. language skills) then we would expect children with CdLS and FXS to show similar levels of implicit task performance to neurotypical children. Finally, we predicted that receptive language ability would be associated with explicit but not implicit task performance in autistic children, and children with CdLS and FXS.

## MATERIALS AND METHODS

### Recruitment

Children were recruited as part of a broader study on the behavioural and cognitive profile of autism characteristics in genetic syndromes; further details of the recruitment are outlined in Ellis and colleagues [31]. Neurotypical children were recruited through advertisements passed on by local schools, whereas autistic children and children with CdLS and FXS were recruited via relevant family support groups, and participant research databases. Neurotypical children were included if they were between the ages four to eight, with an approximate reading age of seven years or below, and were excluded if they had a first-degree relative with a diagnosis of ASD. Autistic children were included if they were aged between four to ten years, had received a clinical diagnosis of ASD and had a history of language delay. Autistic children were excluded if they had a genetic syndrome diagnosis other than CdLS or FXS, with those with CdLS or FXS being included in their respective groups. Children with CdLS and FXS were included if they were aged between four to seventeen years and had received a clinical diagnosis of their respective genetic syndrome. All children were included if they were mobile and could speak at least five words spontaneously and communicatively daily. Only boys with FXS were included due to sex differences in the behavioural phenotype within this group [32–34]. Parents and legal guardians gave written and verbal informed consent for their child. In addition, children who had capacity also gave written and/or verbal consent. The study was granted ethical approval by the University College London Research Ethics Committee (Project ID number: 12763/001) and at the Research Integrity and Governance Office at the University of Surrey (EGA ref: FHMS 19–20 013).

### Participants

The final sample was made up of 34 neurotypical children, 22 autistic children, 9 children with CdLS and 9 children with FXS, who had completed both the implicit and explicit mentalizing tasks. Our initial recruitment target was 20 participants in each group (see [31]); however, data collection was disrupted due to the national lockdown response to the COVID-19 pandemic. It should however be noted that previous work has indicated that even small sample sizes are sufficient to generate large between group effect sizes in genetic syndromes. e.g. [35, 36].

Table 1 reports participants’ demographic information. Groups significantly differed in chronological age, as neurotypical children were significantly younger than children with FXS (*r* = −.41, *p* = 0.009) and children with CdLS (*r* = −.31, *p* = 0.045). However, our recruitment strategy aimed to ensure that groups were comparable on receptive language skills as verbal ability is often associated with social cognitive abilities in neurotypical and autistic children, and children with CdLS and FXS [4, 22]. Groups did not differ on British Picture Vocabulary Scale Third Edition (BPVS3) raw scores [37], with a small effect size (*F*(, 70) = 2.06, *p* = 0.113, ω^2^ = 0.04); however, independent samples t-tests did indicate that neurotypical children had significantly higher scores compared to children with FXS (*t*(41) = 2.18, *p* = 0.035, *r* = 0.10) and CdLS (*t*(41) = 2.408, *p* = 0.021, *r* = 0.11). The effect of these differences in BPVS raw scores were considered where appropriate throughout the analysis (see data analysis section below).

**Table 1:** Participant demographics

	NT (N = 34)	AUT (N = 22)	FXS (N = 9)	CdLS (N = 9)	*p*	Group differences
Mean Chronological age in years (SD)^*^	6.38 (1.17)	7.35 (2.44)	8.85 (2.64)	10.20 (4.96)	.027	NT < FXS, CdLS
Mean BPVS raw score (SD)	96.62 (18.77)	84.27 (35.00)	79.33 (29.06)	76.89 (31.51)	.112	
N female (%)	20 (62.50%)	6 (27.27%)	0 (0%)	6 (66.67%)	.002	NT > AUT, FXS CdLS > FXS
Mean SRS Total T scores	*NA*	91.21 (27.45)^*^	79.75 (7.59)^**^	78.14 (14.28)^***^	.302	

^*^Missing data from three participants ^**^Missing data from one participant, ^***^Missing data from two participants

NT = neurotypical, AUT = autistic

As expected from our recruitment strategy, there were significantly more males in the FXS group than the neurotypical (X^2^ = 9.90, *p* < 0.001) and CdLS groups (X^2^ = 9.00, *p* = 0.009). Whilst the sex distribution between autistic and neurotypical children was significantly different (X^2^ = 4.77, *p* = 0.029), the proportion of male to female autistic participants corresponds with recently reported sex ratios of autism diagnoses [38–40]. There were no significant differences in sex distribution between children with CdLS and neurotypical children, nor children with CdLS and autistic children. No significant differences were found between diagnostic groups in Social Responsiveness Scale t-scores [41], indicating that children with CdLS, FXS and autistic children showed similar levels of autistic characteristics.

### Measures

#### Questionnaires

Parents completed questionnaires about their child. These included a demographics questionnaire, as well as the school-age version of the Social Responsiveness Scale Second Edition (SRS-2; [41]). The SRS-2, suitable for children aged four to 18 years old, was completed by caregivers to assess the presence and frequency of autism-related social and communication characteristics. The SRS-2 was completed only for the autistic children, and children with CdLS and FXS.

#### British picture vocabulary scale third edition (BPVS-3; Dunn, Dunn & Styles, 2009) [37]

The BPVS-3, suitable for children aged between three years to sixteen years 11 months, was used to assess participants’ single word receptive vocabulary and as a proxy of their general language ability. Participants are presented with four pictures and asked to identify the picture that matches a word said by the examiner. The BPVS-3 is suitable for populations associated with ID as it is quick to administer and does not require a verbal response [31]. Raw scores were used, as many participants with an ID (three autistic children, five children with FXS and six with CdLS) did not achieve a raw score high enough relative to their chronological age to be converted to a standard score.

#### Explicit mentalizing tasks

Children completed a range of explicit mentalizing tasks taken from the Theory of Mind Scale [42] and the Sally-Anne task [15]. For the purposes of the current study, a total score was derived from the three tasks assessing false belief understanding; contents false belief also commonly known as the ‘smarties’ task [43], more explicit false belief or ‘Scott’s gloves’ [44], and less explicit false belief tasks or ‘Sally-Anne’ [15] as these were considered most closely matched to the false belief scenario in the implicit mentalizing task. For each task, children were asked a test question that required them to use their understanding of the mental state of the agent in the story to correctly predict the agent’s behaviour (e.g. “where will Scott look for his gloves?”). Children were then asked to justify their choice (e.g. “why will he look there?”) and answer a control question (e.g. ‘Where are his gloves really?) to check children’s overall comprehension of the story. Children could score a total of two points for each task, which were summed to derive a possible maximum total score of six. To receive at least a score of one on a task, children were required to correctly answer both the control and the test questions. If they received this first point, they could then score an additional 1.0 or 0.5 point if they gave a correct justification either with (e.g. because that’s where he thinks they are) or without referencing the characters mental state (e.g. because that’s where he left them) respectively. Higher scores indicated stronger explicit mentalizing abilities.

#### Implicit mentalizing task

**Figure 1:**
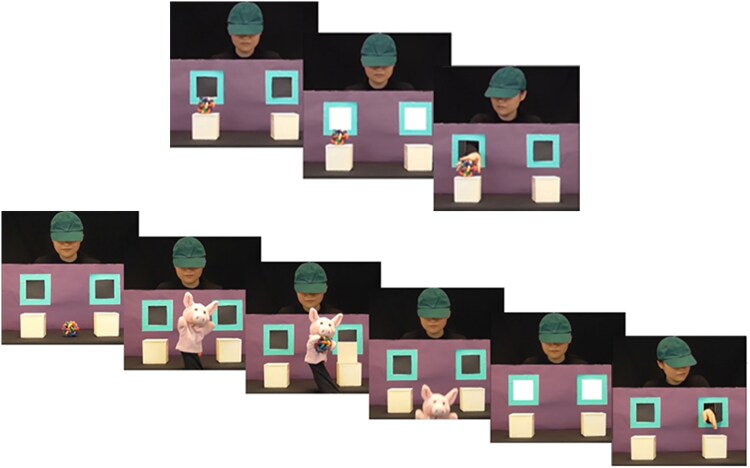
Procedure for short (above) and long (below) familiarisation trials

The implicit mentalizing task is a spontaneous anticipatory looking paradigm using eye-tracking technology, adapted from Senju and colleagues [25] into a multi-trial paradigm following Wu et al. [45]. The paradigm was approximately five minutes long and consisted of four familiarisation trials (two long and two short), three false belief and three true belief trials. Children were given no explicit instructions other than to watch the video. Familiarisation trials (see Fig. 1) were included to help children understand the contingency that the agent in the video would reach for an object that was either to their left or right after a cue (two windows becoming illuminated accompanied by the sound of a chime for 800 ms). Short familiarization trials (six seconds long) began with an object on top of one of the two opaque boxes (left/right), followed by the cue. The agent then reached through the window to retrieve the object. Long familiarization trials (19 seconds long) began with the agent observing a puppet place an object into one of the boxes (left/right boxes and left/right-handed puppeteer), which was followed by the cue, and then the agent reaching through the window to retrieve the object.

**Figure 2:**
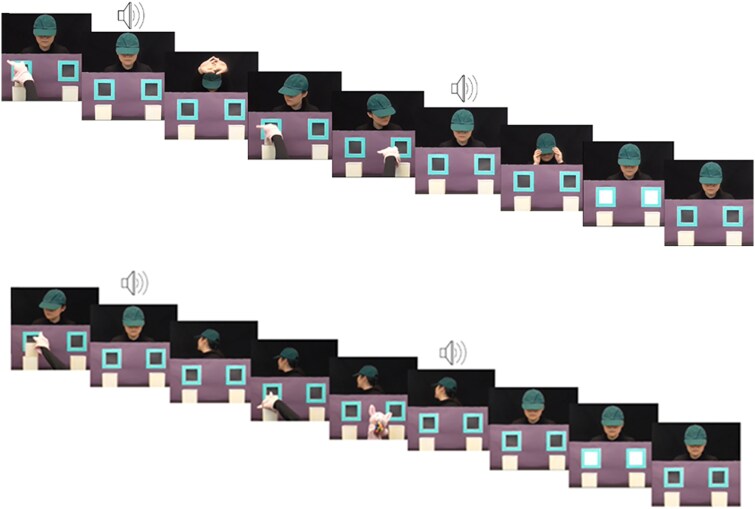
Procedure for true belief (top image) and false belief trials (bottom image)

Following the familiarisation trials, children then watched the experimental block which consisted of three true belief and three false belief trials (randomly selected from a larger set, [45]). Each trial was 36 seconds long (see Fig. 2). The location of the object and the agent’s belief about the object were congruent in the true belief trials, but incongruent in the false belief trials. In the true belief trials, the agent observed the puppet place the object into one of the two boxes and then leave the scene. This was followed by the sound of a buzzer, after which the agent stretched. The agent then observed the puppet removing the object from the original box and moving it to the other box and the puppet leaving the scene, which was followed by the sound of a whistle. The agent then repositioned their hat, which was followed by the cue and the screen paused for four seconds. In the false belief trials, the agent observed the puppet place the object into one of the boxes and leave the scene. This was followed by the sound of a doorbell, after which the agent turned away. The puppet took the object from the box and removed it from the scene. Then there was the sound of a door closing and the agent turned back to face the scene, followed by the cue and pause. The true belief and false belief trials were presented in the same random order for each participant.

***Apparatus***. Stimuli were presented on a 15.6-inch Dell Precision 5520 laptop through Tobii Studio software version 3.4.8 installed, connected to a remote Tobii Pro X3–120 (120 Hz sampling rate) eye tracker. Prior to presentation of the stimuli, children completed a five-point calibration. Videos were filmed and edited using Adobe Premiere pro.

#### Procedure

Children were assessed over one or two sessions either at the University or at their home. Children typically completed the BPVS-3 first, followed by two eye tracking paradigms (the current paradigm and the paradigm outlined in Ellis et al., [31]) and then an imitation task. Some autistic children, children with CdLS and FXS syndrome also took part in the Autism Diagnostic Observation Schedule Second Edition (ADOS-2 [11]). These data are not reported as it was not possible to collect ADOS-2 data for all participants due to the national COVID 19 lockdown occurring during the project. Parents completed questionnaires either during the research visit or in their own time (returned to the research team via post).

#### Data analysis

**Figure 3:**
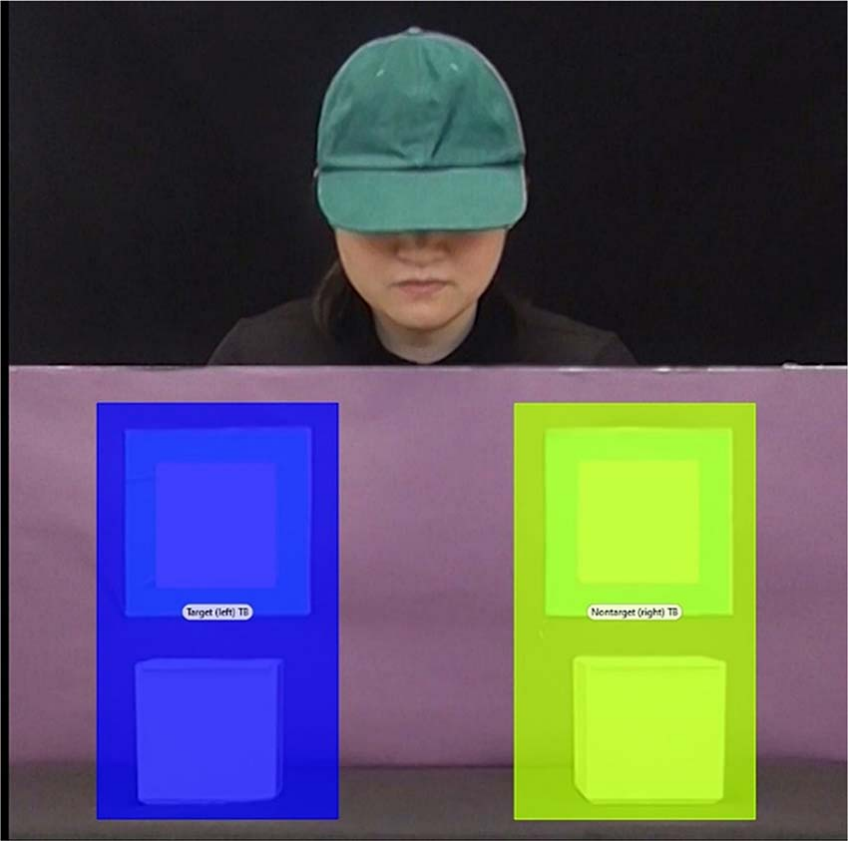
Visual display of AOIs

Areas of interest (AOI) were defined for the area around the left and right box (see Fig. 3). The total time spent fixating within these AOIs from the onset of cue to the end of the trial (a total of 5000 ms per trial) was extracted for the true belief and false belief trials. Total time spent fixating within these AOIs from the onset of the cue and the onset of the agent reaching for the object (a total of 1000 ms) was extracted for the familiarisation trials. Differential looking scores (DLS; [25]) were used to provide a measure for whether participants’ looking was biased towards the target (where the agent thinks the object is) or non-target AOI (the other box) and were calculated for each trial using the following equation:


$$ \frac{( fixation\ duration\ of\ target\ AOI- fixation\ duration\ of\ non- target\ AOI)}{( fixation\ duration\ of\ belief\ congruent\ AOI+ fixation \ duration\ of\ belief\ incongruent\ AOI)}. $$

DLS ranged from −1 (indicating a looking bias towards the non-target AOI) to +1 (looking bias towards the target AOI). A score of 0 indicated that participants looked at both AOIs equally and was considered chance performance. Trials in which participants fixated within neither AOIs during the critical time window were excluded from further analyses. From the remaining trials, mean familiarisation, true belief, and false belief DLSs were calculated for each participant.

Data were analysed using IBM SPSS statistics v29. One sample t-tests or their non-parametric equivalent within groups were run to compare average DLS to chance performance. As some data were non-normally distributed, one-way Quade’s non-parametric ANCOVAs controlling for BPVS raw scores were run to compare explicit mentalizing scores or implicit mentalizing DLSs between groups. Significant main effects were followed up with pairwise comparisons. Kendall-tau correlations were run to investigate the associations between participant’s chronological age or BPVS raw score, with their explicit mentalizing total scores or implicit mentalizing DLS, within each group.

## Results

### Familiarisation trials

To confirm that children in each group understood the contingency that the cue signalled that the agent would reach through one of the windows to retrieve the object, we evaluated whether children showed anticipatory looking in familiarisation trials within each group. Indeed, DLS scores were significantly above chance in the neurotypical (*M* = 0.35, *SD* = 0.43, *t*(33) = 4.690, *p* < 0.001), autistic (*M* = 0.37, *SD* = 0.43, *t*(19) = 4.878, *p* < 0.001, CdLS = *M* = 0.48, *SD* = 0.53, *t*(8) = 2.678, *p* = 0.028) and FXS groups (*M* = 0.38, *SD* = 0.40, *t*(8) = 2.848, *p* = 0.022).

To validate that the paradigm elicited implicit mentalizing, we evaluated whether neurotypical children showed anticipatory looking towards the target AOI in both false belief and true belief trials. Analysis confirmed that neurotypical children’s DLS was significantly above chance in the false belief trials (*M* = 0.22, *SD* = 0.55, t(33) = 2.366, *p* = 0.024) but not the true belief trials (*M* = 0.10, *SD* = 0.39; t(33) = 1.510, *p* = 0.141). Therefore, the following analyses were performed only on the false belief trials.

### Aim 1) performance profiles on explicit and implicit mentalizing tasks in neurotypical and autistic children, and children with FXS and CdLS

**Figure 4:**
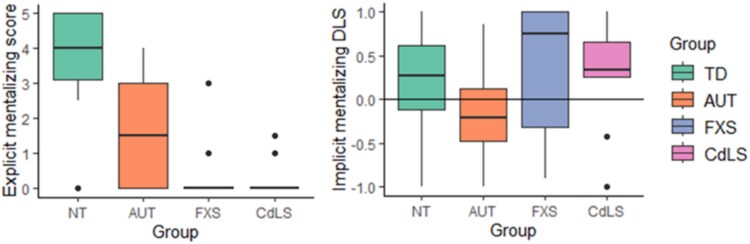
Boxplots of neurotypical children, autistic children, and children with CdLS and FXS explicit false belief scores and implicit DLS

Figure 4 shows the median explicit mentalizing scores and implicit mentalizing DLS for each group. A significant main effect of group was identified for total explicit mentalizing score (*F*(3, 70) = 23.73, *p* < 0.001). Pairwise comparisons revealed that neurotypical children scored significantly higher than autistic children (*t*(70) = 6.19, *p* < 0.001), children with FXS (*t*(70) = 5.93, *p* < 0.001) and children with CdLS (*t*(5.91), *p* < 0.001). No group differences were found between autistic children and children with FXS (*t*(1.37), *p* = 0.186) or CdLS (*t*(1.32), *p* = 0.192), nor between children with FXS and CdLS (*t*(−.02, *p* = 0.988). However, visual inspection of the graphs indicates that there was very little variance in performance within the two syndrome groups, who almost unanimously found the tasks exceptionally difficult.

A significant main effect of group was also identified for implicit mentalizing DLSs (*F*(3,71) = 3.57, *p* = 0.018). These were significantly lower in autistic children relative to neurotypical children (*t*(2.95), *p* = 0.004), children with FXS (*t*(−2.21), *p* = 0.030) and children with CdLS (*t*(−2.15, *p* = 0.035), indicating difficulties on this task only by autistic children. No differences were found between neurotypical children and children with FXS (*t*(−.20, *p* = 0.840) or CdLS (*t*(−.13), *p* = 0.897), nor between children with FXS and children with CdLS (*t*(.06), *p* = 0.956). DLS scores were significantly above chance in the neurotypical group (*z* = 2.25, *p* = 0.024), but not the autistic (*z* = −1.76, *p* = 0.079), FXS (*z* = 1.26, *p* = 0.209) nor CdLS groups (*z* = 1.19, *p* = 0.236). Nevertheless, whereas 14 out of 22 (64%) autistic children had a DLS at or below chance, only two out of 9 (22%) children with CdLS and 12 out of 34 (35%) neurotypical children did so. In children with FXS, four out of nine performed at or below chance (44%). Visual inspection of Fig. 4 indicates that, while implicit mentalizing performance was broadly strong in the FXS group, there was great within-group variation (IQR = 1.54) compared to the other groups (IQR < 0.87).

### Aim 2) associations between chronological age and receptive language ability, and explicit and implicit mentalizing performance

**Figure 5:**
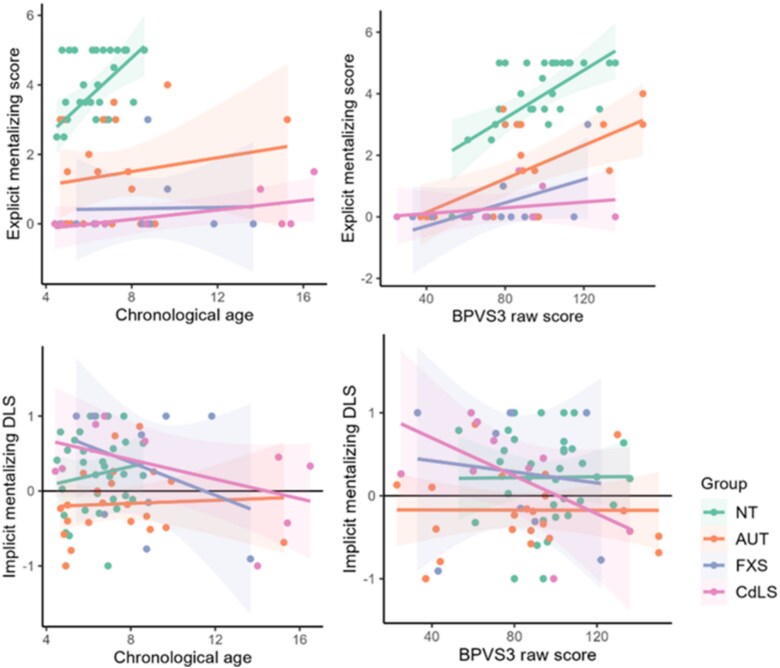
Participants chronological age (in years) and BPVS raw score plotted against their explicit false belief scores and implicit mentalizing DLS

Figure 5 shows participants’ chronological age and BPVS raw score plotted against their explicit mentalizing scores and implicit mentalizing DLS. Table 2 reports the related correlation coefficients.

**Table 2:** Correlation coefficients (with p-values in brackets) for chronological age and BPVS raw scores, and explicit mentalizing scores and implicit mentalizing DLS, for each group

Group	Chronological age	BPVS raw score
Neurotypical		
Explicit mentalizing score	**.37 (.005)**	**.49 (<.001)**
Implicit mentalizing DLS	.14 (.434)	.01 (.962)
Autistic		
Explicit mentalizing score	−.01 (.949)	**.36 (.038)**
Implicit mentalizing DLS	.15 (.338)	−.07 (.672)
FXS		
Explicit mentalizing score	.13 (.664)	.38 (.192)
Implicit mentalizing DLS	−.24 (.381)	−.18 (.511)
CdLS		
Explicit mentalizing score	.47 (.111)	.30 (.311)
Implicit mentalizing DLS	−.51 (.160)	−.57 (.111)

Correlations revealed that both chronological age (τ_b_ = 0.37, *p* = 0.005) and BPVS raw score (τ_b_ = 0.49, *p* < 0.001) were moderately associated with explicit mentalizing scores in neurotypical children. In autistic children, there was a moderate association with BPVS raw score (τ_b_ = 0.36, *p* = 0.038) but not with chronological age. No associations were found between chronological age or BPVS raw score and explicit mentalizing scores in the CdLS and FXS groups. Visual analysis of Fig. 5 demonstrates that this lack of association is due to children with CdLS and FXS scoring at floor level on the explicit mentalizing tasks. Neither chronological age nor receptive language ability correlated with implicit mentalizing DLS in any group.

## Discussion

We present the first study to assess performance on an implicit mentalizing task, and to compare implicit with explicit mentalizing task performance between autistic children, and children with two genetic syndromes (CdLS and FXS) with heterogeneous autism characteristics. Our findings correspond to the previous literature that shows less accurate performance in traditional explicit mentalizing tasks in these groups than neurotypical children [17–19] and more accurate performance on implicit mentalizing tasks in neurotypical compared to autistic children [27]. However, whilst autistic children struggled with both implicit and explicit tasks relative to neurotypical children, the children with FXS and CdLS only struggled with explicit tasks, showing performance comparable to the neurotypical children on the implicit task. This may indicate that spontaneous mentalizing may be a relative strength in these syndrome groups.

The mentalizing ability of children with FXS and CdLS may be masked in traditional explicit tasks as they involve explicit reasoning about others’ mental states and recruit language mechanisms, whereas implicit mentalizing tasks tap into an automatic, unconscious, and rapid processing of others mental states that does not require verbal reasoning [7]. In contrast, traditional mentalizing tasks may mask mentalizing *challenges,* rather than competencies, in some autistic children. The positive association between the explicit mentalizing performance and BPVS raw scores in the neurotypical and autistic children is certainly consistent with previous literature in these groups [30, 46, 47] and supports the interpretation that such explicit tasks recruit language skills as well as conscious mentalistic reasoning. Autistic people typically require a higher verbal mental age than neurotypical children to pass explicit mentalizing tasks, and it has been proposed that these individuals use linguistic reasoning to ‘hack’ solutions to these tasks [48]. This corresponds with the current study’s findings; whilst autistic children with higher BPVS raw scores show better explicit mentalizing performance, their performance is nevertheless delayed relative to their neurotypical peers. The lack of association between our non-verbal implicit mentalizing task and receptive vocabulary however indicates that mentalizing per se is not dependent on such language skills, thus resulting in a more accurate assessment of mentalizing abilities in these populations. This is supported by work showing difficulties on implicit mentalizing tasks, even in autistic adults who perform comparably to neurotypical participants on explicit mentalizing tasks [25, 45].

In the syndrome groups the lack of association between explicit mentalizing performance and BPVS raw scores appeared to be due to most children scoring at floor level on the explicit mentalizing tasks, despite many of these children having the single word receptive language ability to pass these tasks. Eleven of these 18 participants (61%) had a BPVS raw score that corresponds to an age equivalent between five years and 10 years 4 months. Of these, eight children (73%) received an explicit mentalizing score of zero (see supplementary material A). As most neurotypical children are expected to pass these tasks around the age of four years [49], this suggests that other non-linguistic factors may also contribute to difficulties in explicit mentalizing tasks in children with CdLS and FXS. Importantly, the good performance of these groups on the implicit mentalizing task indicates that mentalizing was also not a limiting factor in explicit mentalizing task performance.

Social anxiety in those with CdLS is characterised by either selective mutism (estimated prevalence of 40%; [50]) or reduced levels of verbalisation in social situations [51], which may be a key non-linguistic driver for difficulties on the explicit mentalizing task. Unlike the implicit mentalizing task, participants are expected to interact with and verbally answer an unfamiliar examiner in the explicit tasks. This task reliance on verbal interaction might therefore account for the difficulties evidenced in the CdLS group. However, while verbal interaction is expected in these tasks, two out of three tasks (the ‘Scott’s gloves’ and ‘Sally-Anne’ tasks) could be answered by pointing. While evidencing reduced frequency of verbalisations, people with CdLS show high rates of gesture use [52], arguably a compensatory strategy for difficulties speaking in social situations [53]. As such, difficulties on these explicit mentalizing tasks in children with CdLS may not be entirely due to difficulties surrounding speaking in social settings.

Previous literature has also highlighted the contribution of executive function skills, such as working memory and inhibitory control to explicit mentalizing task performance in neurotypical children [54]. In contrast, our implicit mentalizing task was designed to limit working memory (trials were very short) and inhibitory control demands (the object was removed from the scene, so the child did not need to suppress their own knowledge of its location). There is therefore good reason to think that these factors may have contributed only to explicit mentalizing task performance in our children with CdLS and FXS, supported by existing findings that both groups show difficulties in these abilities relative to mental age matched controls [55–57]. Executive function skills more broadly, may also contribute to the variability observed in explicit mentalizing task performance in autistic children and may also mask mentalizing difficulties in some autistic people [20]. In autistic children aged 4–7, performance on executive function tasks predicted explicit mentalizing outcomes three years later, independent of age, verbal and non-verbal ability, and early explicit mentalizing performance [58]. Whilst future research is needed to determine whether these groups show a dissociation in the way executive function skills relate to implicit and explicit mentalizing, this interpretation supports the notion that the true mentalizing abilities of children with CdLS and FXS, and some autistic children, can be masked by other non-social cognitive abilities.

An alternative explanation for the dissociation between performance on implicit and explicit mentalizing tasks by children with FXS or CdLS, is a dual system approach in which mentalizing can be achieved through either an implicit or an explicit neurocognitive process, supported by both shared and distinct neural mechanisms [7, 59, 60]. Default-interventionist dual-process theories suggest that both implicit and explicit mentalizing share the same core neural mechanism, identified through fMRI studies to include the right temporo-parietal junction (rTPJ) and the medial prefrontal cortex [60, 61]. However, reiterative reprocessing is proposed to move mentalizing processes from being implicit to explicit by building on the initial impressions generated from implicit mentalizing. This is said to be done via an iterative process that integrates lower-level pre-existing learned social knowledge with additional input from a wider range of brain structures to allow more higher order and explicit inferences to be made across a range of contexts [60]. Within the default-interventionist dual-process theory of mentalizing, one may interpret results from the current study as evidence that implicit, automatic and quick mentalizing neurocognitive processes develop in those with CdLS and FXS but the reiterative neurocognitive processes that enable explicit mentalizing do not subsequently develop.

Drawing together these different explanations, it may be the case that these interpretations are not mutually exclusive. Dual process theories lack a clear delineation of the neural basis of the slower, more considered explicit process [62] other than that it recruits additional brain areas or cognitive processes [60]. It is possible that working memory and inhibitory control may be included in these key additional processes. Distinguishing between a masking interpretation (i.e. that non-social cognitive processes mask mentalizing abilities) and the dual process theory interpretation may therefore not be critical for understanding the role mentalizing plays in the specific profiles of autism characteristics in those with CdLS and FXS.

In contrast, autistic children in our study struggled on both implicit and explicit mentalizing tasks, suggesting that the core implicit and automatic neurocognitive processes needed for both implicit and explicit mentalizing develop differently in this group. However, this pattern differs from autistic adults, who show strong performance on explicit tasks but struggle with implicit mentalizing task performance relative to neurotypical controls [25, 45]. One interpretation of these contrasting findings may be that, unlike autistic adults, many of the autistic children in our study had not yet developed the compensatory strategies that enable them to pass explicit mentalizing tasks despite differences in the core neurocognitive processes required for both implicit and explicit mentalizing. Supporting this, autistic adults showed reduced rTPJ activation during both an implicit (participants watched a false belief vignette and afterwards were asked to identify a character’s hat colour) and an explicit (participants were instead asked to identify the character’s belief) false-belief task, despite showing similarly accurate responses to neurotypical adults in both conditions [61].

However, as explicit mentalizing tasks can be passed by using non-social cognitive abilities, this brings to question the validity of explicit tasks in assessing mentalizing in all autistic people, particularly given evidence of a dissociation between explicit mentalizing task performance and autism characteristics in some autistic teenagers [63]. Whilst findings from our study and others [25, 27, 45] suggest that implicit tasks may be a more sensitive measure of mentalizing for autistic people, little is known about whether performance on these tasks are associated with autism characteristics in autistic individuals, as well as those with genetic syndromes associated with heterogenous autism characteristics, such as those with CdLS and FXS. As such, future work comparing how implicit and explicit mentalizing task performance contribute to the distinct profile of autism social-communication characteristics between these populations is needed.

We did not directly assess this relationship between implicit mentalizing task performance and autism characteristics in any of the groups, However, visual analysis of Fig. 4 demonstrates interesting differences between CdLS and FXS regarding the level of variability in implicit mentalizing task performance, providing preliminary insight into whether and how implicit mentalizing task performance may be associated with these groups’ profiles of autistic characteristics. Children with FXS showed wide variability in implicit mentalizing performance, a profile that corresponds with high within-syndrome variability in both the frequency and profile of autistic characteristics observed [64, 65]. For example, on the Autism Diagnostic Interview Revised [66], boys with FXS without co-occurring autism not only show a milder overall presentation of autism characteristics compared to boys with co-occurring autism but also specific social strengths and skills, such as more social smiling [67]. Implicit mentalizing tasks have been proposed as a promising assessment to more sensitively discriminate between autistic and non-autistic people [45] and therefore may also be a potentially sensitive diagnostic tool to distinguish between autistic and non-autistic people with FXS. However, further work is needed with a larger sample of children with FXS to determine whether performance on implicit mentalizing tasks are able to distinguish between those with and without co-occurring autism.

In contrast, despite showing high levels of autism characteristics [2], children with CdLS showed consistently good implicit mentalizing task performance in the current study. This consistent implicit mentalizing performance corresponds with this group’s behavioural profile, characterised by low within-group variability in autism characteristics when controlling for self-help skills [68]. Considering the high rates of social anxiety reported in those CdLS [69] these findings may suggest that other neurocognitive mechanisms associated with social anxiety (e.g. hypervigilance or intolerance of uncertainty; [70, 71]) may contribute to the high levels of autism-related communication characteristics in this group, rather than differences in mentalizing. Future work is needed to compare the relationships between implicit mentalizing, and social anxiety-related neurocognitive profiles. This may help guide whether people with CdLS would benefit from intervention for social anxiety instead of or in addition to autism-related support.

## Conclusion

In this study, we investigated mentalizing performance profiles on an implicit anticipatory-looking false belief task and a battery of traditional explicit false belief tasks in children with CdLS and FXS, and groups of autistic and neurotypical children. Findings indicate that these groups showed evidence of divergent performance patterns on explicit and implicit mentalizing tasks; explicit mentalizing was weak while implicit mentalizing was strong in children with CdLS and FXS. In contrast, both types of mentalizing were difficult for autistic children. These findings indicate there may be a dissociation between implicit and explicit mentalizing performance in children with CdLS and FXS. The association between BPVS raw score and explicit mentalizing task performance in the neurotypical and autistic children suggests that language mechanisms may be recruited for explicit mentalizing task performance. This supports the theory that some autistic people may pass explicit mentalizing tasks through verbal reasoning, rather than mentalizing per se. In contrast, as most children with CdLS and FXS performed at floor level on the explicit mentalizing tasks; despite having the receptive language ability expected to pass, other non-linguistic factors may contribute to difficulties in these groups. Regardless, findings suggest that difficulties in explicit task performance may either mask good spontaneous mentalizing in children with CdLS and FXS, or that these groups develop the automatic and quick implicit mentalizing skills but not the reiterative neurocognitive processes that enable explicit mentalizing. In autistic children, the combination of difficulties with both implicit and explicit tasks suggests that the core implicit and automatic neurocognitive processes needed for both implicit and explicit mentalizing develop differently in this group. The wide variability in implicit mentalizing task performance in children with FXS corresponds with the behavioural variability of autism characteristics observed within this group. Therefore, implicit mentalizing tasks may prove to be a potential sensitive diagnostic tool to distinguish between autistic and non-autistic people with FXS. In contrast, the good performance on implicit mentalizing in children with CdLS indicates that other neurocognitive processes associated with social anxiety may instead contribute to the emergence of high levels of autism characteristics observed in this group. Further work should investigate whether and how implicit and explicit mentalizing task performance is associated with variable profiles of autism social-communication skills in autistic children, and children with CdLS and FXS.

## Limitations and future directions

There are several limitations that need to be considered when interpreting these findings. Although we included true belief trials as a control condition in the implicit task, in which the location of the object and the agent’s belief about the object are congruent, these trials were not included in the analysis as data from the neurotypical participants indicated that this condition did not elicit anticipatory looking. Anecdotally, adults who have taken part in this same paradigm have commented to us that during the true belief trials, they interpreted the agent’s stretch instead as a yawn, wondering if the agent was asleep. The neurotypical children in the current study may have made similar interpretations, indicating that participants may construct a narrative based on social scripts that implicitly uses current contextual and normative knowledge to understand the agent’s actions [72]. Whereas neurotypical children will likely have a social script of the event of a doorbell ringing (as used in the false belief trials), the ‘stretch’ may have created ambiguity of what the narrative of the true belief task was and whether the agent was aware of the object’s location. Other researchers [28] have instead used the familiarisation trials to determine whether children attribute true beliefs. By this logic, as all four groups in the current study showed anticipatory looking in the familiarisation trials, we could conclude that they have demonstrated true belief understanding. Nevertheless, the replication of presence versus absence of anticipatory looking in the neurotypical and autistic children respectively [27] as well as the contrast of patterns in performance between groups provides good evidence that the false belief trial did in fact assess mentalizing abilities.

Another limitation is the small sample size. Our initial target for the study was twenty participants in each syndrome group. However, due to the national lockdown in response to the COVID-19 pandemic, we were unable to collect any further data via the Tobii Pro X3–120 system as this would have required face-to-face visits with participants. Whilst we explored remote webcam eye tracking technology available at that time, piloting in neurotypical children indicated that these methods were not sensitive enough to detect subtle but important social cognitive processes such as mentalizing. Considering the rarity of the syndrome groups and the challenges of collecting data from them, we therefore saw it appropriate to analyze the currently available data. Nevertheless, some analyses may have been underpowered. Whilst children with CdLS and FXS showed implicit mentalizing task performance that was comparable to the neurotypical group and significantly better than autistic children, the time they spent looking towards the target was not significantly above chance. Whilst findings provide early evidence that these children may be skilled at spontaneously mentalizing, future research replicating these findings in larger samples is essential before making any strong conclusions about these abilities in children with CdLS and FXS.

Finally, we can conclude that the performance profiles of these groups in implicit and explicit mentalizing tasks are independent of verbal ability, but we cannot make conclusions regarding non-verbal ability, as children did not take part in a full IQ assessment. We used receptive language ability as a proxy for verbal intelligence, based on existing evidence linking verbal, rather than non-verbal skills to social cognitive abilities in autistic children [22, 30]. In addition, based on previous research findings, we proposed that it is the high language demands of explicit tasks that mask true mentalizing abilities in children with CdLS, FXS and autistic children [17–19, 48]. Nevertheless, future work should investigate whether these profiles of performance on explicit and implicit mentalizing tasks remain after controlling for non-verbal ability in these groups.

## Supplementary Material

supplementary_material_A_kvaf003

Review_history_for_OXFNSC-2025-001_kvaf003_R1_kvaf003

## Data Availability

The conditions of our ethics approval do not permit public archiving of anonymised study data. Readers seeking access to the data should contact Jo Moss (j.moss@surrey.ac.uk). Access will be granted to named individuals in accordance with ethical procedures governing the reuse of clinical data, including completion of a formal data sharing agreement and approval of the local ethics committee.

## Footnotes

1 Whilst autism spectrum disorder (ASD) is the traditional scientific term, we will only use this term when specifically referring to the clinical diagnostic category. Otherwise, we instead use the non-medicalised term autism to respect the language preferences expressed by the broader autistic community (Bottema-Beutel, Kapp, Lester, Sasson & Hand, 2021).
