# Supplementary material for: Evidence of spontaneous mentalizing in children with Cornelia de Lange and fragile X syndromes, but not autistic children
Source: Oxf Open Neurosci. 2025 Oct 23;4:kvaf003. doi: 10.1093/oons/kvaf003 (PMC12648398; doi:10.1093/oons/kvaf003)
Supplement: Review_history_for_OXFNSC-2025-001_kvaf003_R1_kvaf003 [file review_history_for_oxfnsc-2025-001_kvaf003_r1_kvaf003.zip › Review_history_for_OXFNSC-2025-001_kvaf003_R1_kvaf003.odt]

Review history for OXFNSC-2025-001.R1

Round 1 reviews
Reviewer 1
Recommendation – Minor Revision
Comments to the Author

• I propose to modify the manuscript title: the title should involve the concept of mentalizing (or explicit and implicit/spontaneous mentalizing profile) and display at least all clinical groups, not just Cornelia de Lange and Fragile X syndromes.
• In the introduction, he should devote a paragraph to the independent variables of the study, including some specific information on autism and its comparison with Cornelia de Lange and Fragile X syndromes, and give importance to the results found on the autism population.
• I propose to use the term autism spectrum disorder (ASD was very broad, see APA, 2013) instead of autism, the term recommended by scientists and health organizations (DSM-5, APA, 2013, 2022; ICD-11, WHO) and adopted in the literature.
• I believe that the assessment of intelligence (IQ) in this type of research appears important, as it may better contribute to the explanation of some of the results of this study reporting that individuals with Cornelia de Lange and Fragile X syndromes are able to spontaneously represent the false beliefs of others compared to individuals with ASD despite having the same levels of autism. If they don't, these syndromes are considered to have advantages over ASD, since they have the same levels of ASD characteristics.
• I suggest amending the sentence on page 8, line 24: “The final sample was made up of 34 neurotypical children, 22 children with autism, 9 children with CdLS and 9 children with FXS”.
• Deepens the discussion and gives importance to the results found on ASD, as the number of participants with ASD is large compared to other clinical groups.
• Add the title conclusion to Page 26, Line 8.
• It's best to separate the research limitations from the discussion and add them after the conclusion under the proposed title: Limitations and implications or future directions.
Good Luck
Reviewer 2
Recommendation – Minor Revision
Authors compared performance on explicit tasks and an implicit mentalizing task in children with Cornelia de Lange (CdLS), fragile X syndromes (FXS), autism (AUT), with neurotypical (NT) children. Authors report that neither age nor language ability correlated with implicit mentalizing task performance, and children with CdLS and FXS are skilled at spontaneous mentalizing.

NT children’s differential looking scores were significantly above chance in false belief trials (page 16), but there was no report of the same analysis in the FXS or CdLS group. Unless this analysis is significant, the authors may be going too far with “children with CLS and FXS are skilled at spontaneously mentalizing” (p. 1) and “children with CdLS and FXS are skilled at mentalizing” (p. 2), because they test these groups in comparison with the autism group, but don’t seem to report whether they are performing above chance when considered individually. Even if the analysis is significant in both groups, the authors may want to tone down these statements (e.g., “may be skilled at mentalizing”) due to the very small group size.

Given the difference in composition of subject sex, and given the small sample sizes, it is recommended to use mixed-effect regression models to predict mentalizing scores and enter sex as a covariate and age as predictor.

I suggest reformatting the Introduction to not list 4 hypotheses as presently written, but to expand on more general expected outcomes. Currently, the end of the Introduction reads like a grant application, not a manuscript..

Decision letter
Firstly, thank you for your patience- we experienced a difficult time securing reviewers for this manuscript. Your manuscript ID OXFNSC-2025-001 entitled "Children with Cornelia de Lange and Fragile X Syndromes Spontaneously Represent Others' False Beliefs" which you submitted to the Oxford Open Neuroscience, has now been reviewed.  The comments of the reviewer(s) are included at the bottom of this letter.

The reviewer(s) have recommended publication, but also suggest some minor revisions to your manuscript.  Therefore, I invite you to respond to the reviewer(s)' comments and revise your manuscript within the next 30 days of todays date.

Please note that this journal operates with transparent peer review. This means that if your submission is accepted for publication, the full peer review history of your article will publish online alongside your article. This includes reviewer comments, editor decision letters, and your author responses.

To revise your manuscript, log into https://mc.manuscriptcentral.com/oxfnsc and enter your Author Centre, where you will find your manuscript title listed under "Manuscripts with Decisions."  Under "Actions," click on "Create a Revision."  Your manuscript number has been appended to denote a revision.

You may also click the below link to start the revision process (or continue the process if you have already started your revision) for your manuscript. If you use the below link you will not be required to login to ScholarOne Manuscripts.

*** PLEASE NOTE: This is a two-step process. After clicking on the link, you will be directed to a webpage to confirm. ***

https://mc.manuscriptcentral.com/oxfnsc?URL_MASK=48fb3f0b328a4f6abf68d0be8d546b33


You will be unable to make your revisions on the originally submitted version of the manuscript.  Instead, revise your manuscript using a word processing program and save it on your computer.  Please also highlight the changes to your manuscript within the document by using the track changes mode in MS Word or by using bold or colored text.

Once the revised manuscript is prepared, you can upload it and submit it through your Author Centre.

When submitting your revised manuscript, you will be able to respond to the comments made by the reviewer(s) in the space provided.  You can use this space to document any changes you make to the original manuscript.  In order to expedite the processing of the revised manuscript, please be as specific as possible in your response to the reviewer(s).

IMPORTANT:  Your original files are available to you when you upload your revised manuscript.  Please delete any redundant files before completing the submission.

Because we are trying to facilitate timely publication of manuscripts submitted to the Oxford Open Neuroscience, your revised manuscript should be uploaded within the next 30 days from todays date.  If it is not possible for you to submit your revision in a reasonable amount of time, we may have to consider your paper as a new submission.

Once again, thank you for submitting your manuscript to the Oxford Open Neuroscience and I look forward to receiving your revision.


Revision 1 Authors’ Response
Reviewer: 1

Comments to the Author

• I propose to modify the manuscript title: the title should involve the concept of mentalizing (or explicit and implicit/spontaneous mentalizing profile) and display at least all clinical groups, not just Cornelia de Lange and Fragile X syndromes.

We have amended the title to ‘Evidence of Spontaneous Mentalizing in Children with CdLS and FXS, but not Autistic Children’


• In the introduction, he should devote a paragraph to the independent variables of the study, including some specific information on autism and its comparison with Cornelia de Lange and Fragile X syndromes, and give importance to the results found on the autism population.

We thank the reviewer for their suggestion. In response, we have edited the introduction throughout to outline the role of the clinical groups, adding additional information on the rationale for including the autistic group and their comparison with children with Cornelia de Lange (CdLS) and fragile X syndromes (FXS). We have also expanded our discussion of the importance of the study for the autistic community.

Rather than adding a standalone paragraph, we integrated revisions throughout the introduction to help maintain a coherent narrative. These edits complement existing content from the original manuscript, such as the discussion of previous findings showing a dissociation between implicit and explicit mentalizing performance in autistic people (e.g., Senju et al., 2009; 2010) and the potential advantages of implicit over explicit mentalizing tasks in this community (paragraphs 4-6). We also updated the abstract in line with these edits.
The changes are highlighted in the tracked changes document, but key examples include:

• Introducing autism spectrum condition explicitly at the beginning of the introduction.
• Expanding discussion of the importance of comparisons between the autism group and the syndrome groups (e.g. paragraphs 2, 3, & 7).
• Highlighting the importance of understanding where syndrome-specific and non-syndromic autism cognitive profiles may converge or diverge not just for the syndrome groups, but also to autistic people as well (paragraphs 2, 3, & 7). For example, we discuss how autistic children’s performance on mentalizing tasks may provide insight into the mechanisms underlying autistic social and communication characteristics (paragraphs 2 & 3).
• Additional references to previous autism research throughout the introduction

We hope these changes make the rationale for including each clinical group clearer, and the importance of the study for understanding autism both within and outside the context of genetic syndromes associated with heterogeneous autism characteristics.


• I propose to use the term autism spectrum disorder (ASD was very broad, see APA, 2013) instead of autism, the term recommended by scientists and health organizations (DSM-5, APA, 2013, 2022; ICD-11, WHO) and adopted in the literature.

We thank the reviewer for their suggestion. In our original submission, we purposely did not use the term autism spectrum disorder (ASD) out of respect for the autistic community’s preferences, as many have expressed concerns about medicalised and deficit-focused terminology (Bottema-Beutel et al., 2021). However, we appreciate the reviewer’s point about the need for accurate scientific terminology. We have now introduced the term autism spectrum disorder (ASD) and included the following footnote: Whilst autism spectrum disorder (ASD) is the traditional scientific term, we will only use this term when specifically referring to the clinical diagnostic category. Otherwise, we instead use the non-medicalised term autism to respect the language preferences expressed by the broader autistic community (Bottema-Beutel, Kapp, Lester, Sasson & Hand, 2021).


• I believe that the assessment of intelligence (IQ) in this type of research appears important, as it may better contribute to the explanation of some of the results of this study reporting that individuals with Cornelia de Lange and Fragile X syndromes are able to spontaneously represent the false beliefs of others compared to individuals with ASD despite having the same levels of autism. If they don't, these syndromes are considered to have advantages over ASD, since they have the same levels of ASD characteristics.

We thank the reviewer for raising this point. Whilst we did not include a full IQ assessment, we instead used receptive language ability as a proxy for verbal intelligence. This was chosen based on evidence showing associations between verbal, but not non-verbal cognitive skills to social cognitive abilities in autistic children (Happé et al., 1995; Ellis et al., 2020). In addition, previous research suggests that the high language demands of explicit tasks may mask true mentalizing abilities in children with CdLS, FXS and possibly also autistic children (Grant et al., 2007; Ellis., 2018; Cornish et al., 2005; Kimhi, 2014).

Importantly, there were no significant differences in receptive language ability between the autistic, CdLS and FXS groups, and we controlled for language ability in our analyses. Further, although non-significant, the syndrome groups showed slightly lower receptive language scores than the autism group, which would have arguably given them a disadvantage, yet they performed better on the implicit mentalizing task.

Nevertheless, we acknowledge that receptive language is not equivalent to a full IQ assessment, and we have now explicitly noted this as a limitation in the manuscript (see last paragraph of the discussion). Assessing IQ in intellectual disabilities is challenging due to a lack of sensitive but brief cognitive assessments. However, we believe that the current approach allowed us to control for a critical non-social cognitive factor thought to contribute to mentalizing task performance (verbal ability), and our findings suggest syndrome-specific strengths in social cognition independent of language ability and autism characteristics.


• I suggest amending the sentence on page 8, line 24: “The final sample was made up of 34 neurotypical children, 22 children with autism, 9 children with CdLS and 9 children with FXS”.

We have amended the sentence in line with the reviewer’s suggestion, with the exception that we still use identity first language (autistic children) to respect the preferences of the autistic community (Bottema-Beutel et al., 2021).


• Deepens the discussion and gives importance to the results found on ASD, as the number of participants with ASD is large compared to other clinical groups.

We thank the reviewer for their suggestion. In response, we have given greater depth to the discussion concerning the autistic group, as requested.

The changes are highlighted in the tracked changes document, but key examples include:
- Reframing the discussion to position the autistic group as a key group of interest
- A greater emphasis and discussion of autism-related findings throughout, such as:
o A deeper discussion regarding the association between BPVS raw score and implicit and explicit mentalizing scores in autistic children (paragraph 2)
o Discussion of the role of executive function skills on explicit mentalizing task performance in autistic children (paragraph 5)
o A discussion of findings in the autistic group to relevant theoretical frameworks, such as the default-interventionist dual-process theory (paragraphs 8 & 9)
- In addition, additional references to previous autism research and literature have been included throughout the introduction

These revisions now more explicitly highlight the importance of the results found in the autism group in greater detail and depth.


• Add the title conclusion to Page 26, Line 8.

We have now added the subtitle ‘conclusion’ just before the concluding paragraph (paragraph 12) in the discussion section.


• It's best to separate the research limitations from the discussion and add them after the conclusion under the proposed title: Limitations and implications or future directions.

We have edited this in the discussion, as suggested by the reviewer.


Reviewer: 2

Comments to the Author

Authors compared performance on explicit tasks and an implicit mentalizing task in children with Cornelia de Lange (CdLS), fragile X syndromes (FXS), autism (AUT), with neurotypical (NT) children. Authors report that neither age nor language ability correlated with implicit mentalizing task performance, and children with CdLS and FXS are skilled at spontaneous mentalizing.

NT children’s differential looking scores were significantly above chance in false belief trials (page 16), but there was no report of the same analysis in the FXS or CdLS group. Unless this analysis is significant, the authors may be going too far with “children with CLS and FXS are skilled at spontaneously mentalizing” (p. 1) and “children with CdLS and FXS are skilled at mentalizing” (p. 2), because they test these groups in comparison with the autism group, but don’t seem to report whether they are performing above chance when considered individually. Even if the analysis is significant in both groups, the authors may want to tone down these statements (e.g., “may be skilled at mentalizing”) due to the very small group size.

We thank the reviewer for raising this point. We have now reported one sample Wilcoxon signed-rank tests for each group at the end of the analyses addressing aim 1 in the results section. We discuss these non-significant effects in the FXS and CdLS groups in the limitations section. We have also revised our language in line with the reviewer’s suggestion, now stating that children with CdLS and FXS may be skilled at spontaneously mentalizing.


Given the difference in composition of subject sex, and given the small sample sizes, it is recommended to use mixed-effect regression models to predict mentalizing scores and enter sex as a covariate and age as predictor.

We thank the reviewer for their suggestion. Whilst it would be interesting to investigate sex differences, it was not the focus of our study. In addition, there is no within-group sex variance to control for in the FXS group, as only boys were included. This would make it difficult to untangle the effect of FXS as a group versus sex upon mentalizing scores if the sex was controlled for. Finally, the two groups with the most boys showed diverging performance patterns: whereas autistic children showed evidence of struggling with both implicit and explicit tasks relative to neurotypical children, children with FXS only struggled with explicit tasks. Likewise, whereas BPVS raw score was associated with explicit mentalizing task performance in autistic children, no such association was found in the boys with FXS. Therefore, there is good reason to conclude that performance was unlikely to be attributed to sex.


I suggest reformatting the Introduction to not list 4 hypotheses as presently written, but to expand on more general expected outcomes. Currently, the end of the Introduction reads like a grant application, not a manuscript.

We agree with the reviewer and have now amended the introduction in line with their suggestion. We have repeated it below for the reviewer’s convenience.

Based on a vast body of previous research (e.g. Baron-Cohen, 2000; Happé, 1995; Frith, 2001; Grant et al., 2007; Cornish et al., 2005), we expected neurotypical children to show more accurate performance on explicit tasks compared to autistic children and as well as to those with CdLS and FXS. We also predicted that neurotypical children would perform better than autistic children on the implicit anticipatory-looking task, replicating previous findings in this group (Senju et al., 2010; Schuwerk et al., 2016). We expected that performance on the implicit task among children with CdLS and FXS will provide insight into why these groups struggle with explicit mentalizing tasks. If difficulties with explicit task is due to mentalizing difficulties, we would expect children with CdLS and FXS to show less accurate implicit mentalizing task performance compared to neurotypical children. However, if difficulties with explicit performance is due to other task demands (e.g. language skills) then we would expect children with CdLS and FXS to show similar levels of implicit task performance to neurotypical children. Finally, we predicted that receptive language ability would be associated with explicit but not implicit task performance in autistic children, and children with CdLS and FXS.

Revision 1 Reviewer 1 Comments:
I would like to thank the authors of this manuscript for their efforts and for their positive interaction with my comments. I believe that this manuscript should be taken to the next level.
The authors have taken all comments into consideration.
I have only one comment: please reorganize all references (e.g., Lord et al., 2012) according to this journal's guidelines. 

Revision 1 Reviewer 2 Comments:
The authors have addressed my comments well. I only have the remaining request to spell out CdLS and FXS in the title. 

Final Decision letter

It is a pleasure to accept your revised manuscript entitled "Evidence of Spontaneous Mentalizing in Children with CdLS and FXS, but not Autistic Children" in its current form for publication in the Oxford Open Neuroscience.  The comments of the reviewer(s) who reviewed your manuscript are included at the foot of this letter.

Please note that this journal operates with transparent peer review. This means that  the full peer review history of your article will publish online alongside your article. This includes reviewer comments, editor decision letters, and your author responses.

Next steps
You will receive an email from no-reply@scipris.com within roughly one week. This is your invitation to sign up for an account with SciPris, Oxford University Press’ author portal hosted by Aptara. You will need to create an account if you do not already hold one. Please register or log into your account and follow the online instructions which will guide you through signing your licence and paying the APC. The email and the portal have clearly signposted support options if you need any help during this process.

Please note that SciPris is a completely different system from ScholarOne, so your credentials to submit your manuscript here will not work there. Once you’ve created a SciPris account, you will be able to use it whenever you publish with Oxford Open Neuroscience or any OUP journal. Please note that OUP will only ever request payment for applicable fees be made via SciPris or to an OUP bank account. If you ever have concerns about the legitimacy of a request, please do not hesitate to contact a customer services agent via the SciPris portal or directly via oupsupport@scipris.com.

Thank you for your fine contribution.  On behalf of the Editors of the Oxford Open Neuroscience, we look forward to your continued contributions to the Journal.


Reviewer: 1

Comments to the Author
I would like to thank the authors of this manuscript for their efforts and for their positive interaction with my comments. I believe that this manuscript should be taken to the next level.  
The authors have taken all comments into consideration.
I have only one comment: please reorganize all references (e.g., Lord et al., 2012) according to this journal's guidelines.

Reviewer: 2

Comments to the Author
The authors have addressed my comments well. I only have the remaining request to spell out CdLS and FXS in the title.

Associate Editor
Comments to the Author:
Authors have been very responsive to reviews. There remains a couple of comments to address and that is to spell out CdLS and FXS in the title and reformat references. Authors should accept tracked changes and submit a clean copy version with those changes.
